# Direct synthesis and characterization of optically transparent conformal zinc oxide nanocrystalline thin films by rapid thermal plasma CVD

**DOI:** 10.1186/1556-276X-6-568

**Published:** 2011-10-31

**Authors:** Joachim D Pedersen, Heather J Esposito, Kwok Siong Teh

**Affiliations:** 1 School of Engineering, San Francisco State University, San Francisco, CA, USA

**Keywords:** zinc oxide, transparent nanocrystalline film, thermal plasma chemical vapor deposition, annealing, nanorods

## Abstract

We report a rapid, self-catalyzed, solid precursor-based thermal plasma chemical vapor deposition process for depositing a conformal, nonporous, and optically transparent nanocrystalline ZnO thin film at 130 Torr (0.17 atm). Pure solid zinc is inductively heated and melted, followed by ionization by thermal induction argon/oxygen plasma to produce conformal, nonporous nanocrystalline ZnO films at a growth rate of up to 50 nm/min on amorphous and crystalline substrates including Si (100), fused quartz, glass, muscovite, c- and a-plane sapphire (Al_2_O_3_), gold, titanium, and polyimide. X-ray diffraction indicates the grains of as-deposited ZnO to be highly textured, with the fastest growth occurring along the *c*-axis. The individual grains are observed to be faceted by (103) planes which are the slowest growth planes. ZnO nanocrystalline films of nominal thicknesses of 200 nm are deposited at substrate temperatures of 330°C and 160°C on metal/ceramic substrates and polymer substrates, respectively. In addition, 20-nm- and 200-nm-thick films are also deposited on quartz substrates for optical characterization. At optical spectra above 375 nm, the measured optical transmittance of a 200-nm-thick ZnO film is greater than 80%, while that of a 20-nm-thick film is close to 100%. For a 200-nm-thick ZnO film with an average grain size of 100 nm, a four-point probe measurement shows electrical conductivity of up to 910 S/m. Annealing of 200-nm-thick ZnO films in 300 sccm pure argon at temperatures ranging from 750°C to 950°C (at homologous temperatures between 0.46 and 0.54) alters the textures and morphologies of the thin film. Based on scanning electron microscope images, higher annealing temperatures appear to restructure the ZnO nanocrystalline films to form nanorods of ZnO due to a combination of grain boundary diffusion and bulk diffusion.

**PACS: **films and coatings, 81.15.-z; nanocrystalline materials, 81.07.Bc; II-VI semiconductors, 81.05.Dz.

## Background

Zinc oxide [ZnO] is a direct, wide bandgap (E_g _= 3.37 eV at room temperature) semiconductor which has a high exciton binding energy (60 meV) [1-5]. The large bandgap renders pure ZnO to be colorless in appearance and non-absorbing in the visible to infrared wavelengths (optical spectra at and above 375 nm). The high exciton binding energy of ZnO allows excitonic laser action at or above room temperature, in addition to making ZnO the brightest emitter among GaN (26 meV) and ZnSe (20 meV). From an electronic standpoint, ZnO has one of the best conductivities among the transparent conducting oxides [TCO] due to its high charge carrier mobility - ZnO has high experimentally derived electron Hall mobility of up to 200 cm^2^/V-s [6,7] and hole mobilities ranging from 2 to 8 cm^2^/V-s [8,9]. These desirable attributes make ZnO suitable for optoelectronic applications such as transparent thin transistor [10,11], TCO and buffer layers in photovoltaic cells [12,13], light-emitting diode [8,9], UV laser [14], optical waveguide [15], and biochemical sensors [16]. In spite of these desirable attributes, most current methods of synthesizing ZnO thin films - including plasma enhanced chemical vapor deposition [CVD] [17], thermal CVD [18], radio frequency [RF] or DC magnetron sputtering [19-21], metal organic chemical vapor deposition [MOCVD] [22], spray pyrolysis [23], pulsed laser deposition [24], thermal evaporation [25], hydrothermal [26], and sol-gel processes [27] - often require substantial vacuum, expensive consumables (e.g., diethyl zinc, dimethyl zinc, ZnO sputter target), catalyst (e.g., gold), and lengthy synthesis time. While solution-based methods - such as hydrothermal and sol-gel - can produce good quality films [28] at a much lower processing temperature (approximately 100°C) that are favorable to mass production, vapor phase methods such as thermal evaporation and MOCVD provide important alternative routes to produce high quality films. Nevertheless, in addition to the high vacuum (10^-4 ^to approximately 10^-5 ^Torrs) required, the high temperature at which these vapor phase methods are performed (800°C and above) also makes the process not CMOS-compatible. Therefore, a direct, rapid, close-to-ambient pressure vapor phase synthesis method using inexpensive precursors is highly desirable from a synthesis and process development standpoint.

To address such challenges, this paper reports a rapid, direct, self-catalyzed thermal plasma chemical CVD process for depositing a conformal, nonporous nanocrystalline ZnO thin film on various crystalline and amorphous substrates using solid zinc as the precursor material at 130 Torr. Thermal plasmas - high power discharges - can be produced at or near ambient pressure using high-power sources, such as RF induction plasma system [29]. Previous research has shown that inductive heating can provide a useful and efficient means to rapidly introduce a large amount of heat for nanomaterial synthesis [30-32]. This is attributed to the high enthalpy of RF induction plasma and its being capable of high-frequency (13.56 MHz) switching, making it well suited for applications where high-temperature and high-heating rate heat treatments are needed [33]. In particular, RF induction plasma systems have shown an industry-scale utility for synthesis of high-quality nanoparticles [33]. In thermal induction plasma nanoparticle synthesis methods, concurrent introduction of complex liquid, gas, or powder precursors enables a one-step, cost-effective, and time-efficient synthesis. During synthesis, the reagents are introduced into a plasma-entrained flow, become fully ionized, and condense as droplets as they leave the plasma region. In addition to nanoparticle synthesis, thermal plasma CVD has also found success in ZnO thin film synthesis at a subatmospheric pressure using gaseous precursors such as diethyl zinc or dimethyl zinc [34-36]. While diethyl zinc has been the gaseous precursor of choice, it is expensive, toxic, and pyrophoric and requires special care in handling. Using an environmentally benign precursor is therefore highly desirable. To date, little has been done using solid zinc as the precursor in thermal induction CVD due to the higher temperature typically required in creating Zn vapor. In this paper, we introduce a thermal plasma CVD process using only solid zinc as the source material, thereby simplifying the design of the synthesis system. We demonstrate the deposition of conformal, nanocrystalline ZnO films that are electrically conductive and optically transmissive.

## Experimental details

### ZnO thin film synthesis

**Figure 1:**
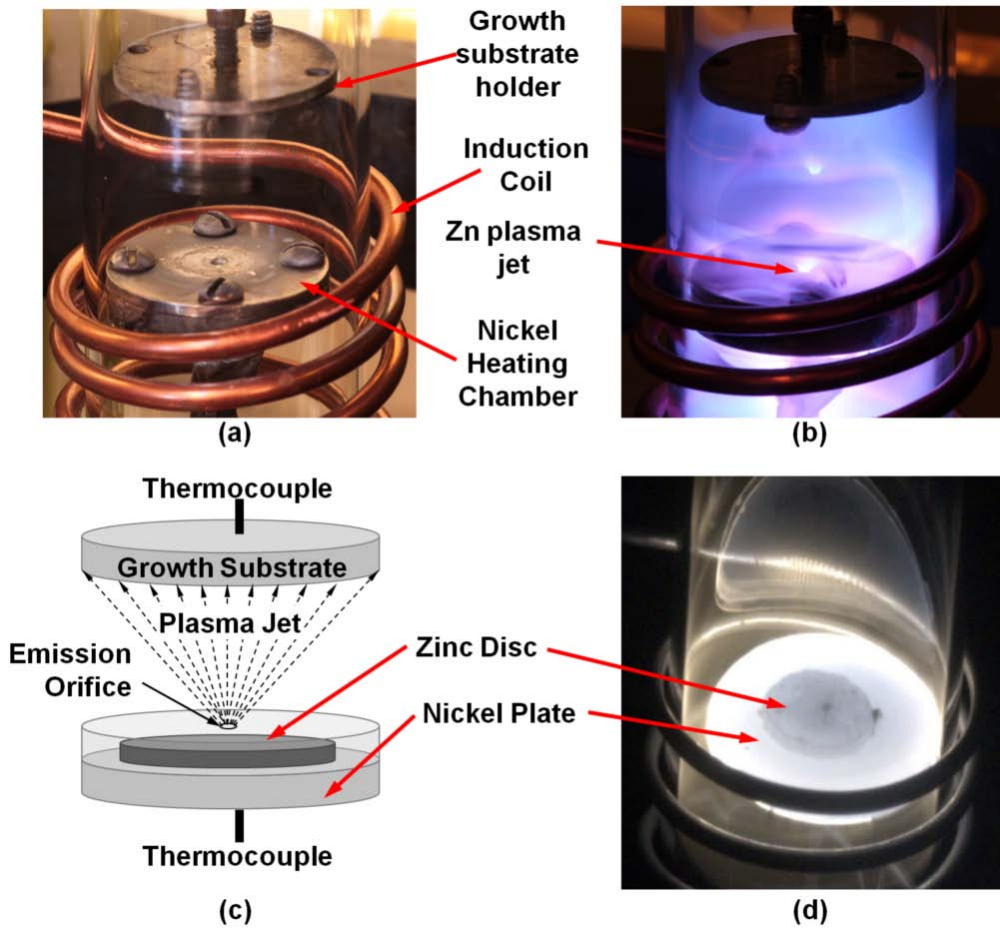
**ZnO nanocrystalline film synthesis system**. Thermal plasma chemical vapor deposition system for depositing ZnO nanocrystalline thin films which consists of a 13.56-MHz RF generator and a matching network, induction coil, zinc source (nickel heating chamber), and substrate holder. (**a**) The synthesis chamber showing the position of the nickel heating chamber in relation to the induction coil. (**b**) When RF is activated, the nickel heating chamber is inductively heated by Joule heating and by the inductively coupled argon/oxygen plasma. Molten zinc is bombarded by high-energy argon, producing zinc ions that are ejected from the emission orifice. Subsequently, zinc vapor reacts with oxygen to form ZnO, which deposits on the growth substrate. (**c**) Anatomy of the synthesis setup showing the location of the solid zinc disc that is enclosed within a nickel heating chamber and the formation of a plasma jet. (**d**) Infrared image of an exposed nickel chamber showing uniform temperature distribution across both nickel and zinc.

Synthesis of ZnO is performed in a quartz process tube at a base pressure of 130 Torr as shown in Figure 1. The inductive heating synthesis system consists of a 13.56-MHz 600-W signal generator (MKS Instruments, Andover, MA, USA), an adjustable auto-matching network configured for an inductive load, a 40-mm-diameter quartz process tube, and a process tube support that has built-in cooling air vents. Two main components of the synthesis system - the source and growth substrates - are contained within the sealed quartz chamber and flushed with argon and oxygen at a ratio of 99.67% to 0.33% at a total flow rate of 301 sccm. The source is made up of solid zinc (99.999% purity; Strem Chemicals, Inc., Newburyport, MA, USA) contained within a pure nickel heating chamber. The top side of the nickel heating chamber is perforated to create an orifice that acts as the Zn emission source. As RF power is turned on, the induced magnetic field, by virtue of the coils, produces (1) Joule and hysteresis heating - up to nickel's Curie temperature of 358°C - in the nickel chamber, and (2) inductively coupled argon-oxygen plasma. Zinc melts in the crucible and ionizes before being ejected from the emission orifice in the form of a Zn plasma jet (Figure 1b, c). It is noted that no Zn plasma is detected before the melting point of Zn (420°C) is reached. The infrared image in Figure 1d shows the uniformity of the temperature distribution of both the exposed solid zinc and the supporting bottom plate of the nickel heating chamber. As Zn ions leave the orifice and are transported toward the fringe of the plasma, they react with oxygen in the synthesis chamber to form ZnO nanoparticles. These nanoparticles that are formed in-flight supersaturate in the boundary layer of the growth substrate and deposit on the growth substrate surface as ZnO nuclei, forming the foundation for subsequent deposition of ZnO nanocrystalline films. The source temperature attained by the nickel heating chamber is 570°C, and the corresponding deposition temperature experienced by the growth substrate ranges from 160°C to 330°C.

We deposit ZnO on crystalline and amorphous growth substrates including p-type silicon (100), mica (muscovite), fused quartz, c- and a-plane sapphire, borosilicate glass, tin-doped indium oxide [ITO], and polyimide (Kapton^®^, DuPont, Wilmington, DE, USA). The deposition rate (10 to 50 nm/min) is tightly controlled by a closed-loop temperature control algorithm where the output RF power is modulated by the source temperature. Based on experience, for a high rate of deposition, the RF power and plasma intensity - which is proportional to the rate of change of the temperature of the nickel heating chamber - must be relatively high, yet the nickel heating chamber temperature is to be maintained well below the boiling point of Zn, so that Zn droplets do not form and deposit on the substrate as metallic zinc. This is achieved by maintaining the temperature of the nickel heating chamber using a saw-toothed temperature profile to attenuate the power periodically. The controller RF output is pulsed to high power to maintain the appropriate nickel heating chamber temperature rate increase. As an upper temperature limit is reached, RF power is automatically reduced allowing the nickel heating chamber to cool to a predetermined temperature. Further pulses can be programmed until the zinc source is completely depleted. At the end of the deposition run, oxygen gas is switched off, and the system is allowed to cool down to room temperature under only Ar gas flow at the original flow rate.

### Post-process film treatment and characterization

The surface morphology, film thickness, and crystal dimensions of the synthesized ZnO nanocrystalline films are characterized by scanning electron microscopy [SEM] on a Zeiss Ultra 55 (Carl Zeiss Microscopy, Peabody, MA, USA) that is equipped with a Schottky field emission gun. Elemental analysis is conducted using an Oxford energy dispersive X-ray probe. Film crystallinity is investigated using an X-ray diffractometer (Bruker D8 ADVANCE, Bruker AXS Inc., Madison, WI, USA) with Cu-Kα radiation (*λ *= 1.54178 Å) and a scanning range of 2*θ *between 24° and 100°. Electrical conductivity measurement is conducted using a four-point probe, and transmittance of the as-deposited film is measured using a Lambda UV-Vis spectrophotometer (PerkinElmer, Inc., Waltham, MA, USA) with an integrating sphere. The spectra are collected in the 200- to 800-nm spectral range. Thermal annealing of samples is performed in a tube furnace (MTI GSL-1100X, MTI Corporation, Richmond, CA, USA) at 300 sccm of argon flow at temperatures ranging from 750°C to 950°C for 1 h.

## Results and discussion

### Properties of as-deposited ZnO film

**Figure 2:**
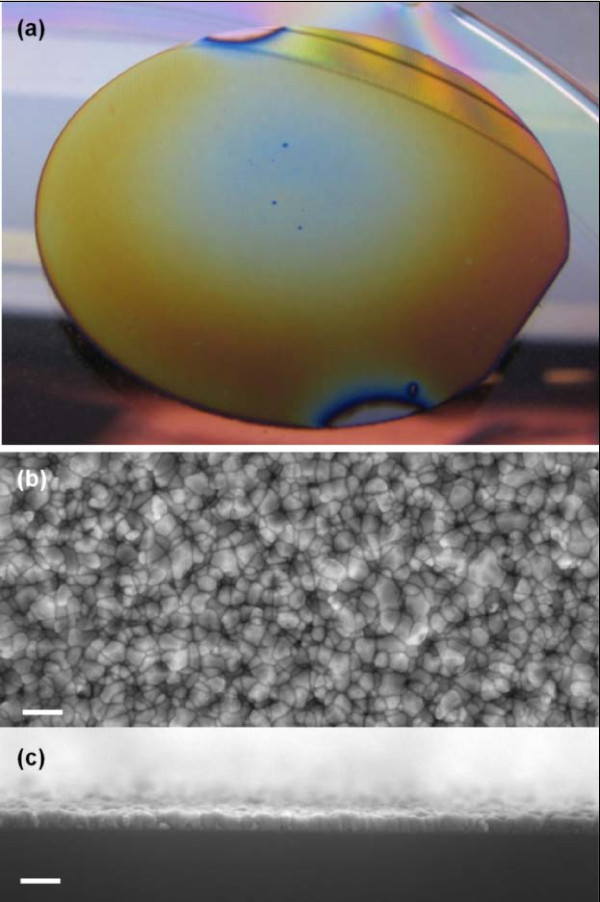
**Optical and SEM images of ZnO film deposited on p-type Si(100)**. (**a**) ZnO nanocrystalline thin film as-deposited on p-type Si(100). (**b**) SEM image (top view) and (**c**) SEM image (edge-on view) of ZnO nanocrystalline thin film showing uniformity and nonporous nature of the film.

**Figure 3:**
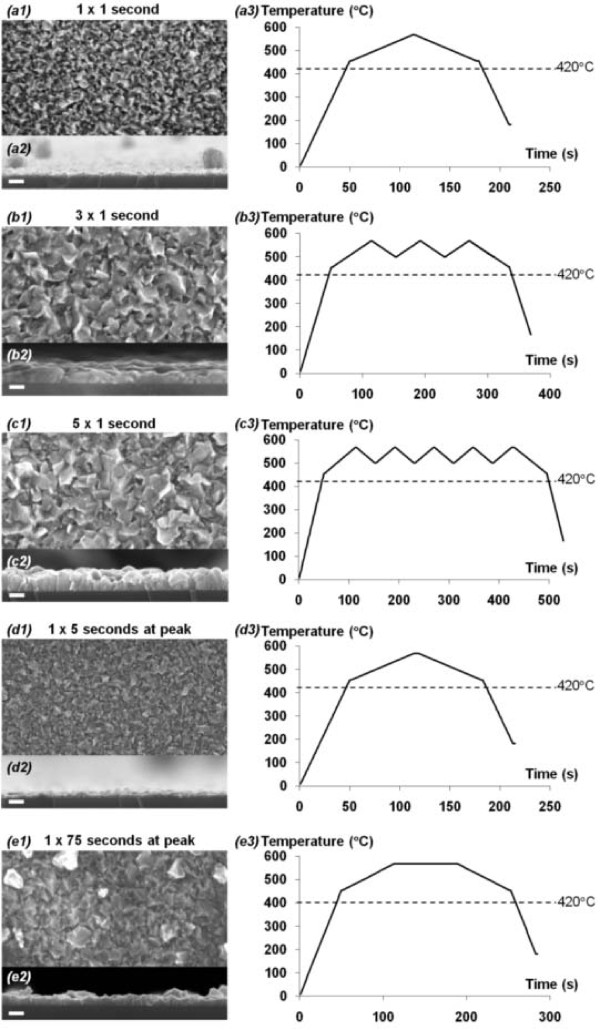
**SEM images of ZnO films deposited using different source temperature profiles**. (**a1-e1**) SEMs (top view), (**a2-e2**) SEMs (edge-on view), and (**a3-e3**) source temperature profiles of ZnO nanocrystalline films deposited with saw-toothed temperature profiles that have resident times of (**a3**) 135 s, (**b3**) 290 s, and (**c3**) 445 s above the melting point of zinc (420°C). The corresponding nominal thicknesses are (**a2**) 25 nm, (**b2**) 70 nm, and (**c2**) 108 nm. (**d**) and (**e**) show the SEM images of ZnO films deposited using saw-toothed temperature profile similar to (**a**) but with longer times - (**d3**) 5 s and (**e3**) 75 s - at the peak temperature of 570°C. The thicknesses of films hence deposited are (**d2**) 22 nm and (**e2**) 57 nm. Scale bar = 100 nm.

At a constant argon-to-oxygen (99.67% to 0.33%) ratio and a constant total flow rate (301 sccm), the morphological and dimensional properties of the ZnO nanocrystalline thin films are found to be dependent on factors including source temperature profile, deposition duration above the melting point of zinc (420°C), substrate type and temperature, and thermal annealing temperatures. Figure 2 shows the optical image and SEM images (top view and edge-on view) of a typical ZnO film deposited on a p-type silicon (100) using a saw-toothed symmetric heating profile (Figure 3), where the rates of heating and cooling are identical. As shown, the ZnO film deposited on the p-type silicon (100) surface appears to be highly uniform.

### Influence of source temperature profile and growth duration

**Figure 4:**
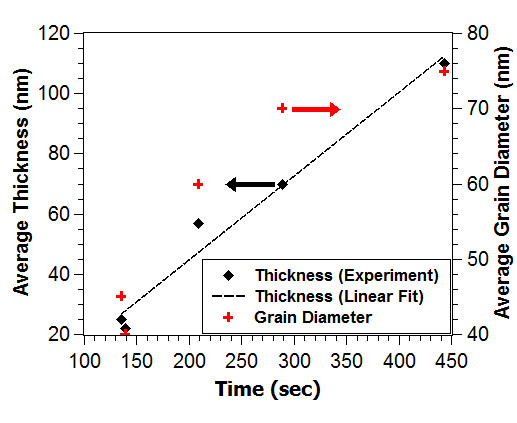
**Relationships between film thickness, grain diameter, and deposition time**. At a source temperature at or above 420°C, the ZnO film thickness scales linearly with deposition time at a growth rate of 16.7 nm/min. Grain diameter also increases as deposition duration increases.

We find clear evidence in the experimental data that correlates the total duration of the Zn source heated at and above the melting temperature of zinc (420°C) in the heating chamber to the as-deposited film thicknesses, grain sizes, and grain structures. SEM images of ZnO deposited from a nickel heating chamber subjected to saw-toothed temperature profile up to 570°C show a strong positive linear relationship between the heating durations above 420°C and the film thicknesses, as shown in Figures 3 and 4.

As the number of saw-tooth ('pulse') and the total synthesis duration above 420°C increase, the nominal thicknesses of the films increase proportionately from 25 nm (1 pulse, 135 s, Figure 3a) to 70 nm (3 pulses, 290 s, Figure 3b), and 110 nm (5 pulses, 445s, Figure 3c) at a growth rate of 16.7 nm/min. We also investigated the influence of the resident time that the source temperature stays at the peak temperature (570°C) on film thickness. We compare two samples, Figure 3a, d, where each has an identical saw-toothed heating profile. The sample in Figure 3a has 1 s of resident time at 570°C, while that of Figure 3d has 5 s of resident time at 570°C. Our results show that there is no significant difference in the thicknesses between these two samples, and the differences are within the range of errors - the sample in Figure 3a has a nominal thickness of 25 nm, while the sample in Figure 3d has a nominal thickness of 22 nm. As shown, there are no noticeable differences in the thicknesses despite the fact that the sample in Figure 3d has four more seconds at the peak temperature. This shows that the total duration the source temperature stays above 420°C, instead of at the peak temperature of 570°C, plays a more critical role in influencing the thicknesses of the films. Furthermore, when comparing Figures 3 d and e, the effect of the duration the source stays above 420°C becomes more obvious - longer synthesis time above 420°C leads to thickening of the film (to 57 nm) as shown in Figure 3e, the sample which is exposed to 70 s longer than the sample in Figure 3d. It is evident that film thickness is predominantly influenced by the heating duration at or above the melting point of zinc and, to a minimal extent, by changes in the resident time at the peak temperature. The evidence indicates that heating to just above 420°C is sufficient to allow deposition to occur in the argon-plasma environment. The grain sizes also appear to increase from an average grain diameter of 44 nm to 75 nm as synthesis duration increases from 135 s to 445 s.

### Influence of substrate type and substrate temperature

**Figure 5:**
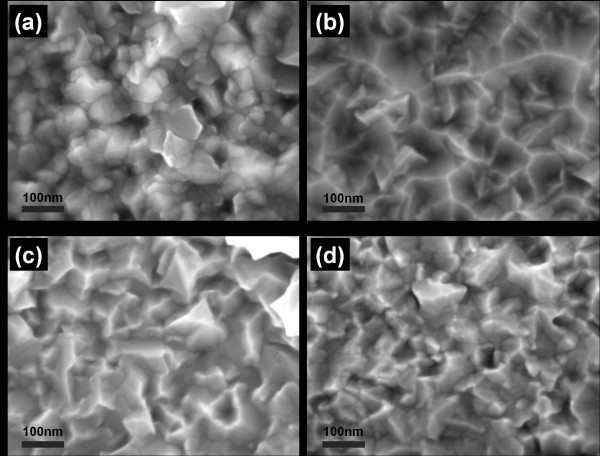
**Morphologies of ZnO films deposited on ceramic and metal substrates**. The morphologies of ZnO films deposited on (**a**) p-type Si(100), (**b**) muscovite, (**c**) gold, and (**d**) titanium at a substrate temperature of 330°C.

We have deposited ZnO nanocrystalline films on various substrates including crystalline p-Si (100), c-plane sapphire, and a-plane sapphire; amorphous fused quartz; borosilicate glass; muscovite; gold; titanium; and polyimide (Kapton^®^, DuPont, Wilmington, DE, USA). Figure 5 shows ZnO nanocrystalline films as deposited on four ceramic and metallic substrates at substrate temperatures of 330°C. The morphologies of ZnO films deposited appear to be largely deposition surface-independent for these substrates as the substrate materials remain chemically and structurally stable at the growth conditions and process temperature of 330°C. As shown in Figure 5a, b, c, d, film coverage on the ceramic and metallic substrates appears to be substrate-independent: the films are continuous with no observed porosity. Close examination of the films' cross sections under SEM reveal no evidence of epitaxial growth of ZnO on any of these substrates and in particular, crystalline a-plane sapphire, which has the closest lattice match with the c-plane of ZnO among all these substrates.

**Figure 6:**
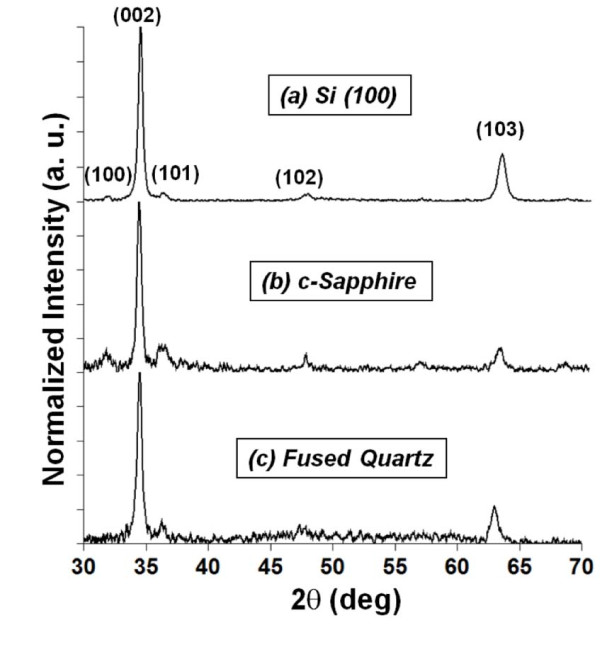
**XRD of ZnO films deposited on ceramic substrates**. Normalized XRD of ZnO films deposited on (**a**) p-type Si(100), (**b**) c-plane sapphire, and (**c**) fused quartz at substrate temperature of 330°C.

Using an extended structural zone model proposed by Mahieu et al. [37], the growth of ZnO film at a homologous temperature of 0.27 (at 330°C) follows a surface diffusion-limited Volmer-Weber island growth model. At this homologous temperature, ZnO nuclei that form initially will grow into small grains with faceted structures due to self-surface diffusion of adparticles on the underlying nuclei and grains. The anisotropic growth rates of different crystallographic planes also dictate the morphology of the grain structures. In the case of ZnO grains, (002) plane has the lowest surface energy, and hence, the growth rate of a ZnO grain is highest in the direction perpendicular to the (002) plane [2,28,38]. Faceting of the ZnO grain is terminated by the planes of the slowest crystallographic growth rate, which as indicated by Figure 5, is the (103) plane. This is confirmed by Figure 6 which shows the normalized X-ray diffractographs of ZnO films deposited on p-type Si(100) and c-plane Al_2_O_3_, and fused quartz to be (002) plane-dominated. Each of these films demonstrates a strong preferential orientation in the (002) plane direction. The next dominant growth is in the direction perpendicular to the (103) planes. The ratios of the relative intensity peaks of (002)/(103) planes for all substrates are consistently between 4 to approximately 7. SEM shows the presence of (103) peaks to be largely attributed to the terminating face, (103) plane, of the ZnO crystal. Peaks attributed to (100), (101), and (102) are also present but are small compared to the (002) peak.

**Figure 7:**
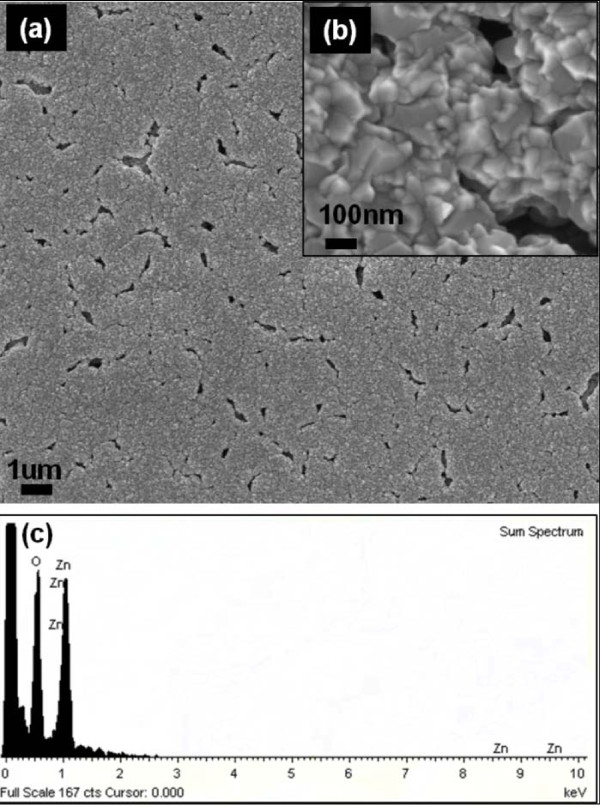
**SEM and EDX images of ZnO film deposited on polymer**. (**a, b**) SEM images of ZnO nanocrystalline film deposited on polyimide (Kapton^®^) and (**c**) EDX of the ZnO film.

For ZnO film deposition on polyimide, the substrate temperature was controlled at 160°C, the lowest deposition achievable with this system. ZnO films deposited on polyimide at 160°C appear to be conformal, as shown in Figure 7a, b, yet contain microcracks and or voids as observed under the SEM. The as-deposited ZnO is conductive enough that SEM imaging can be achieved without an additional conductive coating. Energy dispersive X-ray spectroscopy confirms the presence of ZnO on polyimide in Figure 7c. The microcracks and voids on as-deposited ZnO films are attributed to a large thermal mismatch between ZnO (5 to 8 × 10^-6^/°C) and the underlying polyimide substrate (35 to 40 × 10^-6^/°C). During the temperature ramp-up phase, the polyimide substrate continues to expand while ZnO is being deposited. This imposes a biaxial tensile stress on the ZnO once a continuous ZnO film has formed. As a result, as subsequent ZnO is being deposited, the underlying ZnO continues to be subjected to more tension from the polyimide substrate until the tension is released by the formation of microcracks and voids. The dimensional expansion reaches its maximum value at the highest deposition temperature of 160°C. It is hypothesized that during the subsequent cooling down stage, some of these cracks are closed due to a larger shrinkage of the polyimide vis-a-vis the ZnO film.

### Influence of thermal annealing

**Figure 8:**
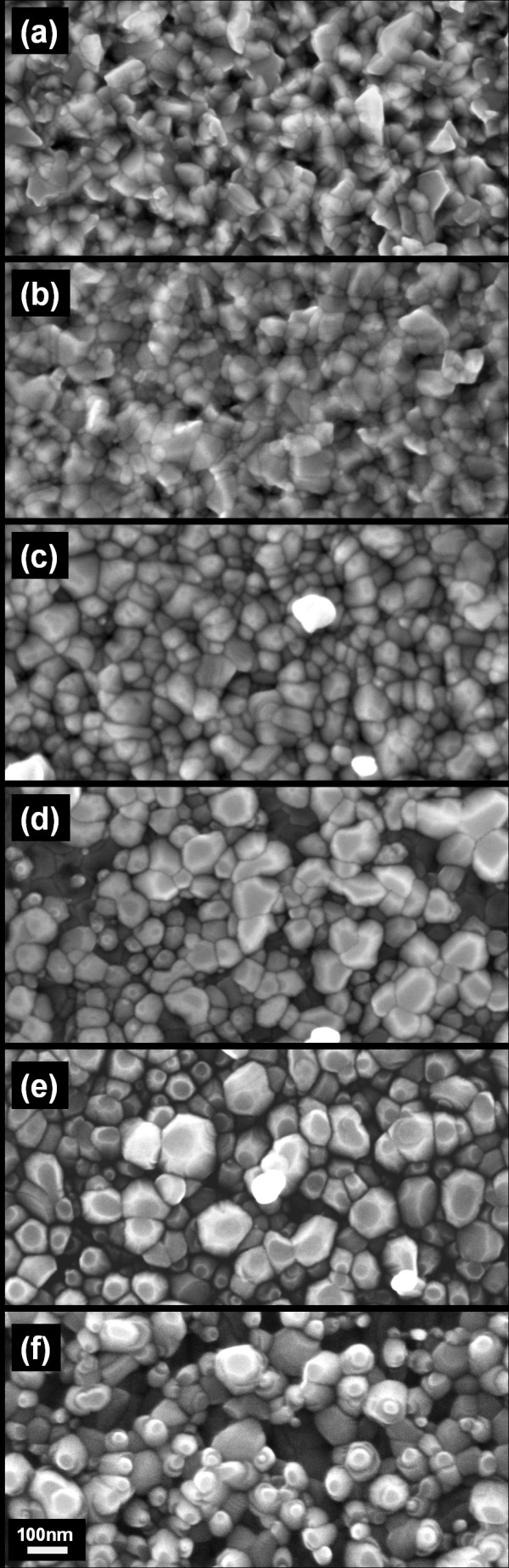
**SEM of ZnO films annealed in pure argon at temperatures from 750°C to 950°C**. Annealed samples from films of initially identical morphology and average grain sizes show an increasing restructuring of film texture with higher annealing temperatures. (**a**) As-deposited ZnO film and ZnO films annealed at (**b**) 750°C, (**c**) 800°C, (**d**) 850°C, (**e**) 900°C, and (**f**) 950°C. Scale bar = 100 nm.

Thermal annealing in pure argon environment at temperatures ranging up to 950°C is performed to elucidate the effect of heat treatment in the absence of oxygen on the grain morphologies and dimensionalities of ZnO grains. Five cleaved specimens of 200-nm-thick ZnO films deposited on one p-type Si (100) wafer are annealed for 90 min at various temperatures ranging from 750°C to 950°C in a tube furnace supplied with 300 sccm argon at 130 Torr. SEM image (Figure 8) shows definitive morphological changes that are correlated to annealing temperatures. Annealing at 750°C (0.46 T_m_) results in the growth of ZnO grains into larger grains with greater definition at the grain boundaries. At 800°C (0.48 T_m_), restructuring of the grain texture produces conspicuous (002) facets along with increased grain sizes and lower grain density. As annealing temperature increases from 800°C to 900°C (0.52 T_m_), the increasing thermal energy input causes further surface texture restructuring due to grain boundary diffusion and bulk diffusion. This in turn accelerates grain growth in the direction perpendicular to the (002) plane - the ZnO plane with the lowest surface energy - and produces columnar ZnO grains that continue to elongate along the *c*-axis. Accompanying this change is the noticeable growth in the direction parallel to the surface of the substrate, i.e., in direction normal to (100) planes. During grain growth, the larger grains are formed by consuming smaller adjacent grains, which lowers the grain density. The columnar ZnO crystals would act as seeds for the seeded growth of nanorods as temperature is further increased to 950°C (0.54 T_m_). SEM images show that at 950°C, nascent nanorods form on the aligned ZnO nanocrystals. At the same time, it is also observed that the grain density continues to decrease from 900°C to 950°C. This is likely due to the increased bulk diffusion that provides for the growth of the nanorods.

### Optical properties

**Figure 9:**
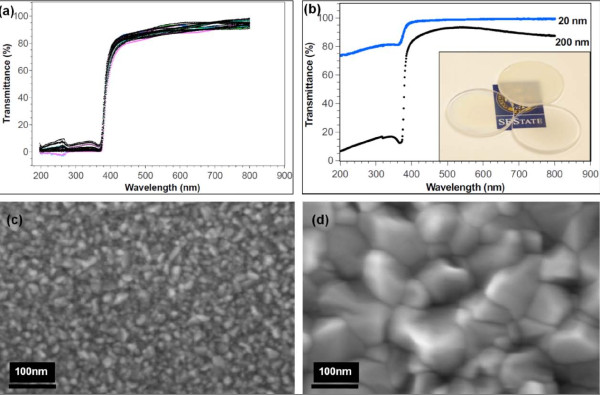
**Optical properties and grain structure of 20-nm- and 200-nm-thick ZnO films**. (**a**) Optical transmittance of 25 samples of 200-nm-thick ZnO film deposited on borosilicate glass slides. (**b**) Optical transmittance of 20-nm- and 200-nm-thick ZnO films deposited on 1.6-mm-thick fused quartz wafers. (inset) Clockwise from top: 1, 000-nm-, 200-nm-, and 20-nm-thick ZnO films on fused quartz. SEM images of (**c**) 20-nm- and (**d**) 200-nm-thick ZnO films deposited on fused quartz.

Figure 9 shows the optical properties of ZnO nanocrystalline films deposited on borosilicate glass substrates and fused quartz. Figure 9a shows the optical transmittance spectra of 200-nm-thick ZnO deposited under identical process conditions on 25 samples of borosilicate squares (25.4 mm × 25.4 mm). The optical transmittance spectra are measured in the wavelength range of *λ *= 200 nm to 800 nm. The optical characteristics of these samples show that the system is producing films of consistent quality, thickness, and uniformity. The high repeatability and deposition consistency of our process minimizes run-to-run variation in terms of film thickness and optical quality. Figure 9b shows the UV-Vis spectrum of a ZnO film at two thicknesses, 20 nm and 200 nm, deposited on fused quartz. At both thicknesses, sharp UV absorption edges at around 375 nm are observed, which corresponds to an optical bandgap energy, *E*, of approximately 3.26 eV according Equations 1 and 2 [39,40]:

(1) E=ℏω−(α(ω)⋅ℏωB)1n

(2) α(ω)=2.303log10(1T)d

where *E *is the optical bandgap, *ħω *is the photon energy in eV, *α*(*ω*) is the absorption coefficient, *ω *is the angular frequency, *B *is a constant between 10^5 ^and 10^6 ^cm^-1^, *T *is the transmittance, *d *is the film thickness, and *n *is an exponential value = 1/2 [39]. The optical bandgap energy for our films is closest to films deposited by spray pyrolysis (3.26 eV) [41] and close to films deposited by other methods such as CVD (3.19 to 3.23 eV) [42] and pulsed laser deposition (3.26 eV) [43] and (3.1 eV) [44]. As shown in Figure 9b and the inset, ZnO films at 20 nm and 200 nm exhibit high transmittance in the visible range; however, the transmittance below 375 nm depends largely on the film thickness - thinner films appear to be more transmissive, while thicker films are less. Figure 9c, d shows the grain morphologies of the 20-nm- and 200-nm-thick ZnO films, respectively. The average grain sizes correspond to 25 nm and 100 nm, respectively, for the 20-nm and 200-nm films. Finally, for the 200-nm-thick ZnO film, the four-point probe measurement shows a conductivity of up to 910 S/m, indicating the ZnO film to be highly conductive.

## Conclusions

We have successfully demonstrated a direct, catalyst-free synthesis method of depositing conformal nanocrystalline ZnO films on crystalline and amorphous substrates using a fast thermal plasma CVD process that is preceded by inductive heating. SEM indicates that the ZnO films deposited on ceramic and metal substrates at 330°C are highly conformal with evenly distributed grain sizes and a preferential growth direction along the *c*-axis. ZnO films deposited at 160°C on polyimide are conformal; however, due to a large thermal mismatch between the film and the substrate, stress-induced porosity is observed. Such porosity is expected to be reduced with a lower substrate temperature. Optical measurements of 20-nm- and 200-nm-thick ZnO films show a high optical transmittance at spectra 375 nm and above, which corresponds to the optical bandgap of ZnO. Thermal annealing of ZnO films at temperatures ranging from 750°C to 950°C causes restructuring of the grain. As annealing temperature increases, grain boundary diffusion and bulk diffusion cause restructuring of ZnO grains into ZnO nanorods.

## Abbreviations

CVD: chemical vapor deposition; EDX: energy dispersive X-ray; ITO: tin-doped indium oxide; SEM: scanning electron microscopy; TCO: transparent conducting oxides; UV-Vis: ultraviolet-visible light; XRD: X-ray diffraction; ZnO: zinc oxide.

## Competing interests

The authors declare that they have no competing interests.

## Authors' contributions

JDP and KST built the synthesis system and instrumented a closed-loop temperature control. JDP and HJE carried out the experiments and data collection. KST and JDP interpreted the results together. KST performed data analyses and prepared the manuscript.
